# What Have Advances in Transcriptomic Technologies Taught us About Human White Matter Pathologies?

**DOI:** 10.3389/fncel.2020.00238

**Published:** 2020-08-04

**Authors:** Sarah Jäkel, Anna Williams

**Affiliations:** Centre for Regenerative Medicine, Institute for Regeneration and Repair, University of Edinburgh, Edinburgh, United Kingdom

**Keywords:** single-cell transcriptomics, single-nuclei transcriptomics, human neuropathology, white matter, multiple sclerosis, RNA-sequencing, “omics” approaches

## Abstract

For a long time, post-mortem analysis of human brain pathologies has been purely descriptive, limiting insight into the pathological mechanisms. However, starting in the early 2000s, next-generation sequencing (NGS) and the routine application of bulk RNA-sequencing and microarray technologies have revolutionized the usefulness of post-mortem human brain tissue. This has allowed many studies to provide novel mechanistic insights into certain brain pathologies, albeit at a still unsatisfying resolution, with masking of lowly expressed genes and regulatory elements in different cell types. The recent rapid evolution of single-cell technologies has now allowed researchers to shed light on human pathologies at a previously unreached resolution revealing further insights into pathological mechanisms that will open the way for the development of new strategies for therapies. In this review article, we will give an overview of the incremental information that single-cell technologies have given us for human white matter (WM) pathologies, summarize which single-cell technologies are available, and speculate where these novel approaches may lead us for pathological assessment in the future.

## Introduction

### Classical Approaches to Study Human White Matter Pathology

Modern neuropathology has its origins in the late 19th and early 20th century when famous neurologists or psychologists such as Santiago Ramon y Cajal, Jean-Martin Charcot, and Alois Alzheimer started to describe and illustrate the central nervous system (CNS) and its pathological changes. These early, but still accurate and detailed illustrations of the brain and individual cells, were all based on histological stains observed through a simple light microscope. It took many years before pathology could reach another level of detail with the common use of antibodies to develop marker-specific immunological stains that are still state-of-the-art in modern research laboratories. Due to the combination with fluorescent labels and the development of better microscopes, this method has become a standard technique to study human pathology and it has helped us to gain a deep understanding of cellular and sub-cellular structures of the brain in health and disease.

Multiple Sclerosis (MS), a chronic inflammatory and demyelinating neurodegenerative disease of the CNS, is a good example of how this descriptive pathology is still used, but it equally applies to other pathologies. The characteristic lesions in white matter (WM) tracts can be classified into active, chronic active, chronic inactive, and remyelinated lesions—so-called shadow plaques (Lassmann et al., 1998). This still highly used classification system is based on the presence of demyelination and the distribution of infiltrating immune cells in and around the lesions and is carried out with simple histological staining on post-mortem human tissue. So far, there are only limited ways of detecting the different lesion stages during the lifetime of a patient using non-invasive imaging techniques (Brück et al., 1997; Hemond and Bakshi, 2018). Specific magnetic resonance imaging sequences enable the detection of chronic active lesions where acute inflammation is happening at the lesion rim (Absinta et al., 2018) and these are associated with disability and ongoing tissue damage (Absinta et al., 2019) aiding prognosis. These new imaging paradigms are exciting but we are still far from a full picture of MS lesions either by pathology or live imaging. Moreover, we still have limited knowledge about molecular or mechanistic changes in MS lesions which are key to understanding the disease.

With the development of new technologies in the 2000s, many labs started to use bulk RNA-sequencing (RNA-seq) or DNA-based microarrays to describe cellular and molecular changes in disease at the transcript level, to gain a deeper insight into functional pathological changes. This was the beginning of a revolution in pathology, helping define molecular markers of disease and raising new hypotheses for disease pathogenesis, to be tested experimentally. This revolution continues with new methods to identify transcripts from single cells or nuclei and to identify these transcripts spatially on the tissue. Here, we describe this revolution, and how this is evolving and will impact our understanding of human WM pathology. Although these techniques apply to a wide range of human WM CNS pathologies, this review will mostly use MS as an exemplar and only touch on other diseases where relevant.

## Modern Approaches to Study WM Pathologies

### What Have We Learned From Whole Transcriptomic Approaches?

**Figure 1:**
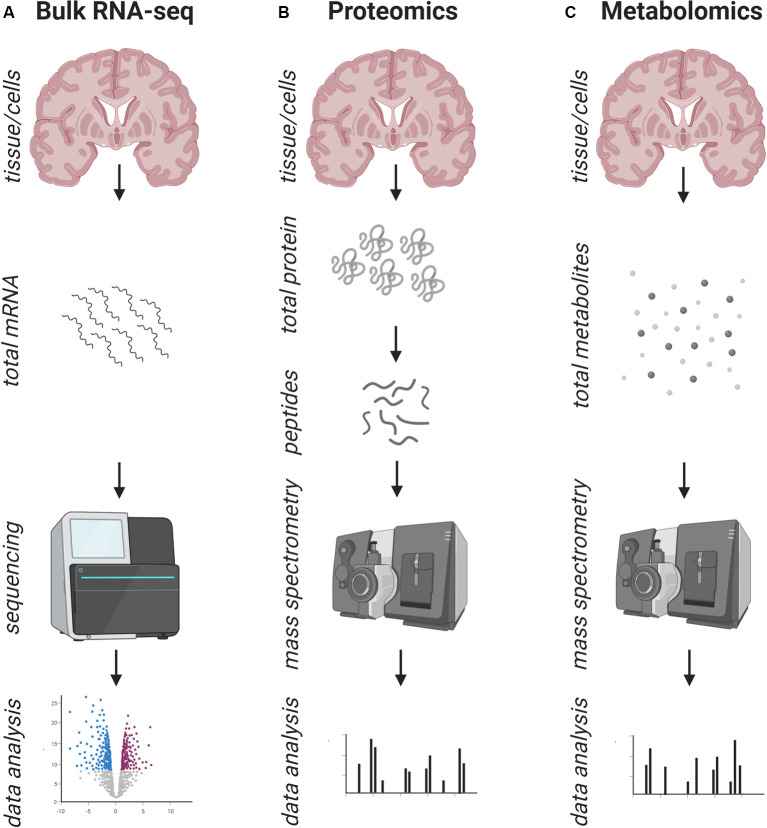
Schematic overview of the workflow of common bulk approaches to address human pathology. **(A)** Standard workflow of bulk RNA-sequencing: the entirety of mRNA/RNA is isolated from a tissue/cell type of interest and prepared for next-generation sequencing (NGS), which is followed by bioinformatics data analysis. **(B)** Standard workflow of bulk proteomics: the entirety of proteins is isolated from a tissue/cell type of interest and prepared for mass spectrometry, which is followed by bioinformatics data analysis. **(C)** Standard workflow of bulk metabolomics: the entirety of metabolites is isolated from a tissue/cell type of interest and prepared for mass spectrometry, which is followed by bioinformatics data analysis. In all approaches **(A–C)** the information about the cell of origin and the spatial distribution is lost.

Bulk RNA-seq is a method to detect the entirety of the transcriptome within a sample of interest, which can either be a whole piece of tissue or sorted cells from a tissue. This can be done in an unbiased way where RNA is isolated, fragments transcribed into cDNA, which is further linked with specific adapters making them compatible with next-generation sequencing (NGS), which is then bioinformatically analyzed (Figure 1A). Alternatively, in a more biased way, isolated RNA is loaded onto specific microarray chips containing probes for only predefined gene transcripts. Commercial microarrays contain a large number of probes for the most important gene transcripts spread over the whole genome so that it can still be relatively unbiased. However, early experiments also included home-made arrays with lower numbers of genes.

The hallmark of WM MS pathology is the clearly distinguishable focal demyelinated lesions where myelin is lost. Therefore, due to its ease of detection and separation from the surrounding normal-appearing white matter (NAWM), many groups have performed bulk transcriptome studies on MS tissue comparing these. Besides, probably in part due to its easier accessibility at least in life, many have been performed on body fluids such as blood or cerebrospinal fluid (CSF) but some also used whole-brain or spinal cord tissue samples. Several review articles have already summarized these comparisons and discussed the technical challenges (Comabella and Martin, 2007; Kinter et al., 2008; Dutta and Trapp, 2012). In summary (Figure 4), the major findings are that all analyzed tissue sources express high numbers of inflammatory gene transcripts, although the inflammatory pathways differ. Also, it became clear that NAWM is not equal to control WM, suggesting that MS is a more global disease than previously thought. Many other findings of transcript differences across these studies were unique to a specific gene transcript in one study rather than having common ground between studies, providing interesting candidates to be investigated. Most surprisingly and against the common concept in MS that oligodendrocytes are the primary target of the attack, it has been suggested that surviving oligodendrocytes around demyelinating lesions in the NAWM are induced by hypoxia to be neuroprotective and anti-inflammatory and are thus more actively involved in disease and perhaps limiting it (Graumann et al., 2003; Zeis et al., 2008). Very recently, heparan sulfate production by mature oligodendrocytes around demyelinating lesions is one of the mechanisms in limiting demyelinating lesions (Macchi et al., 2020). Lindberg et al. (2004) came to similar conclusions regarding the NAWM and additionally pointed out that the immune response activation is different in the different compartments with a more cellular response in NAWM and a humoral response in lesions. Looking a bit closer into differences between demyelinated lesions, Tajouri et al. (2003) found that although both acute and chronic lesions share the majority of markers that are changed in MS in comparison to control, the fold change of those gene transcripts is however quite different. More recent publications have used bulk RNA-seq on MS tissue in a more complex way to explore either transcriptomic changes of microglia in the initial phase of MS (van der Poel et al., 2019) or transcriptomic changes in a hormonal context of the hypothalamus-pituitary-adrenaline (HPA)-axis (Melief et al., 2019). The first study reported an increase in transcripts related to lipid metabolism in microglia sorted from NAWM that is similar to those found in active demyelinated lesions, however, whilst maintaining their homeostatic functions. The latter study found that gene expression networks in MS tissue correlate with the activity of the HPA axis and/or disease severity, showing that gene expression in a pathological context is not only regulated by the pathology itself but also depends on other environmental factors. Thus, careful consideration of the experimental design and the case selection must be part of planning such an experiment.

**Figure 2:**
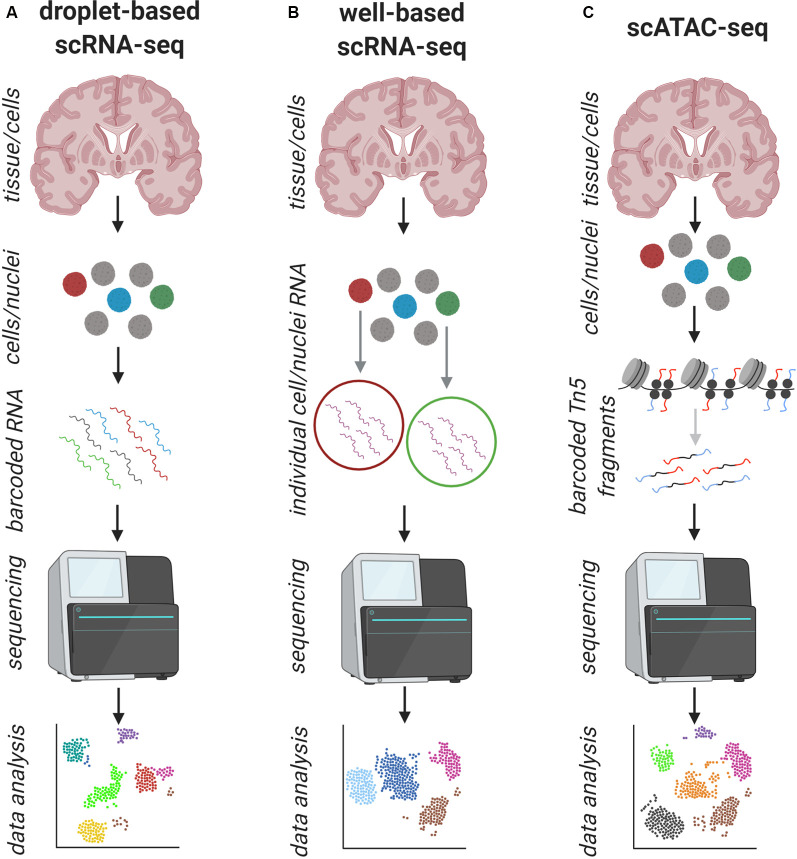
Schematic overview of the workflow of common single-cell/nuclei (sc/sn) approaches to address human pathology. **(A)** Standard workflow of droplet-based sc/sn RNA-sequencing: tissue is dissociated into a single-cell/nuclei suspension and the mRNA of each cell is captured and barcoded individually and prepared for next-generation sequencing (NGS), which is followed by bioinformatics data analysis. This can be done for up to ten thousands of cells. **(B)** Standard workflow of well-based sc/sn RNA-sequencing: tissue is dissociated into a single-cell/nuclei suspension and the cells are captured in individual wells where each cell is prepared for NGS, which is followed by bioinformatics data analysis. This is usually done for less than 1,000 cells. **(C)** Standard workflow of droplet-based Assay for Transposase-Accessible Chromatin using sequencing (ATAC-seq): tissue is dissociated into a single-cell/nuclei suspension and the open chromatin regions of each cell are cut and barcoded individually and prepared for NGS, which is followed by bioinformatics data analysis. This can be done for up to ten thousands of cells. In all approaches **(A–C)** the information about the cell of origin is maintained, but the spatial distribution is lost.

**Figure 3:**
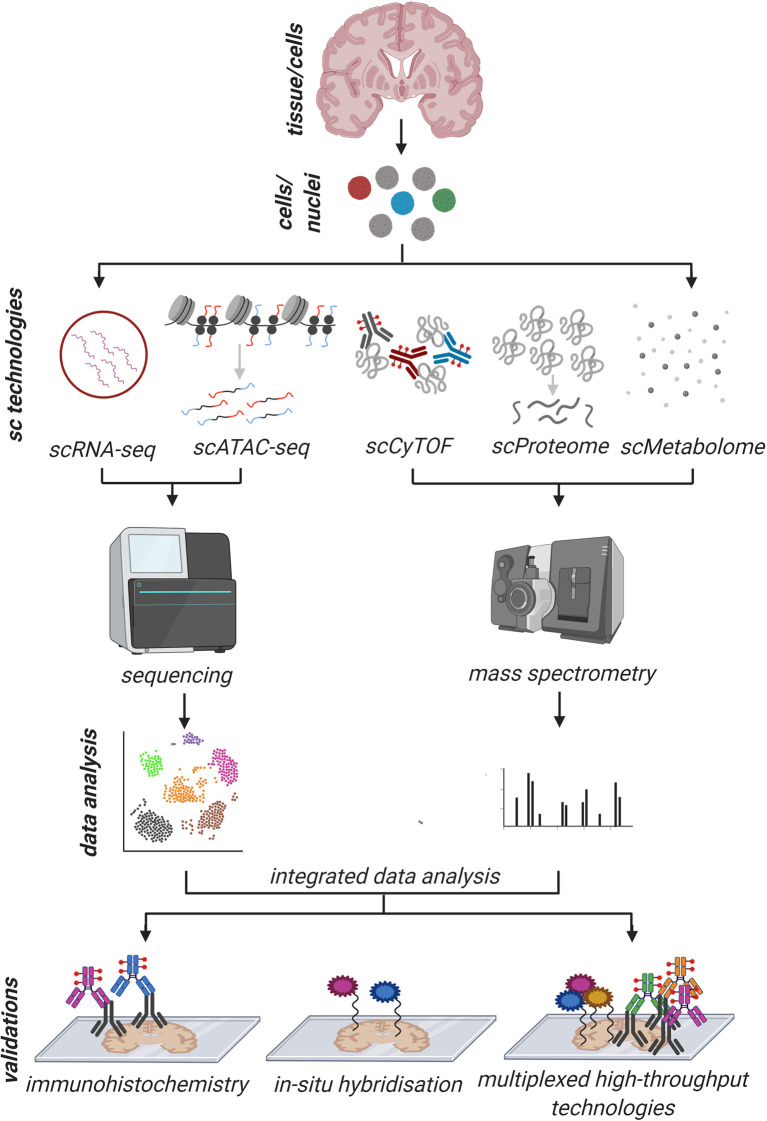
Schematic overview of an imaginative holistic workflow of common single-cell/nuclei (sc/sn) and validation approaches to address human pathology. The tissue is dissociated into a single-cell/nuclei suspension and sc/snRNA-seq, sc/snATAC-seq, scCyTOF, scProteomics, and scMetabolomics are performed in parallel on the same tissue source with their respective workflows. After individual and comparative/integrated bioinformatics data analysis, the results are validated, ideally, on a different tissue source by standard immunohistochemistry (IHC), *in situ* hybridization (ISH) and other high throughput multiplexed techniques (100–1,000 genes/proteins of interest) where IHC and ISH can be combined. With such a possible workflow, first, the information about the cell of origin is maintained, and with the validation techniques, the spatial resolution can be analyzed as well. This holistic approach would allow us to thoroughly exploring human pathology from different angles to gain deeper information.

**Figure 4:**
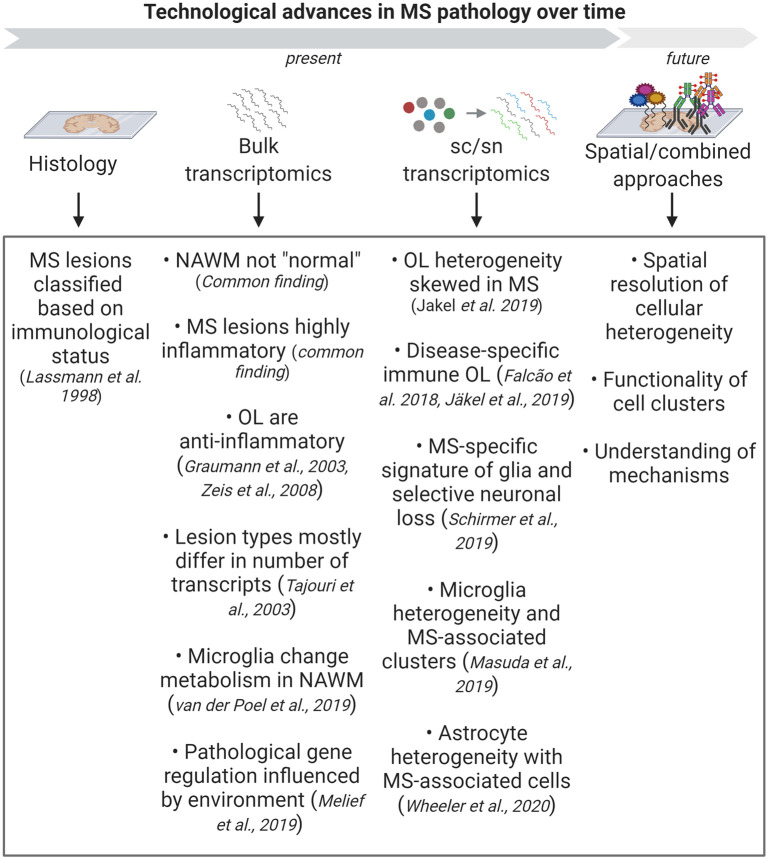
Summary of the biological findings in Multiple Sclerosis (MS) that were gained through advances in technology. Classical histology has helped to classify MS lesions based on their immunological status. The development of bulk transcriptomic approaches has helped to unravel many transcriptional pathways that are changed in MS, with the most important common theme being inflammation. More recent studies using sc/snRNA-seq has started to unravel cellular heterogeneity and changes in the representation of these cells in disease. Combining different sc/sn approaches and using spatial technologies in the future will help to deepen our understanding of the functionality of the heterogeneous clusters and the underlying pathological mechanisms.

Bulk transcriptomic approaches have brought several advantages to the field, but as ever with evolving technology, also some challenges. In contrast to immunohistochemistry (IHC) or quantitative PCR (qPCR) studies of candidate genes, it is unbiased, or relatively unbiased (with microarrays) allowing detection of new mechanisms rather than only digging deeper into already known ones. It is also not dependent on good primers/antibodies or experimenter choice. Long interfering non-coding RNAs are a good example of this, as most of their roles are relatively understudied and one specific RNA was found to play an important region-specific role in a study on Multiple System Atrophy (MSA), another human WM neuropathology, suggesting regional differences of this RNA to control brain function (Mills et al., 2015). Another advantage, at least in theory, is that studies that are performed by different groups in different tissues should be easy to compare, as all capture RNA in an unbiased way. However, disadvantages are plentiful, limiting comparisons as early studies (at least) used low numbers of individuals as input and findings might thus not be representative for a larger MS cohort. Comparisons chosen have varied and have included: (1) Lesions vs. NAWM (Whitney et al., 1999, 2001; Tajouri et al., 2003); (2) Lesions/NAWM vs. Controls (Graumann et al., 2003; Lindberg et al., 2004; Zeis et al., 2008, 2018); and (3) different lesions and/or different regions of lesions (Lock et al., 2002; Mycko et al., 2003; Hendrickx et al., 2017). Furthermore, MS lesions can occur in all WM regions and transcriptional profiling may be different when the lesions from the different studies come from different regions, for example from cerebellar WM and frontal subcortical WM. Many of these studies used non-standardized RNA isolation methods, different types of microarrays (commercial and homemade) with different probe sets (quantity and type), and also different sensitivities for lowly abundant genes, which may explain why different studies found so many different results. Highly abundant genes may mask more subtle effects, and in MS, this often leads to the discovery that MS lesions are associated with demyelination and inflammation (Kinter et al., 2008)—not quite a surprise for the inflammatory demyelinating disease.

A further disadvantage is that bulk transcriptomic studies detect gene expression irrespective of their cellular source within the tissue, so that a signal may be lost if one gene transcript is significantly upregulated in one cell type, but downregulated in another. This becomes especially important when studying a tissue with little cellular heterogeneity, as bulk approaches are generally able to detect a shift in cell-type proportions (e.g., more inflammatory cells in MS lesions), but are less good in detecting changes within similar cells sharing the majority of transcripts.

### How Can Complementary Bulk Approaches Help Address WM Pathologies?

Other technologies, such as proteomics and metabolomics may also illuminate human pathologies (Figures 1B,C). They are either suitable to help validate hypotheses generated by transcriptomics or to generate new hypotheses themselves. Proteomic approaches using different methods of mass spectrometry have been widely used (as summarized in Farias et al., 2014; Farias and Santos, 2015). Numerous studies have been performed on human blood and CSF samples, which are easier to obtain, and may allow the development of new disease biomarkers in living patients (Del Boccio et al., 2016) as well as elucidating potential mechanisms of pathogenesis. The scarcity of reliable blood or CSF biomarkers for MS has been quite sobering to date, which might also be due to the technical challenges of highly abundant proteins (e.g., albumin) in the samples that mask smaller changes. Few proteomic studies so far have been performed on human brain tissue itself (Han et al., 2008; Broadwater et al., 2011; Ly et al., 2011), perhaps as a full proteomic overview of isolated brain tissue is technically challenging. This is due to the high abundance of proteins that cannot be captured by current technologies, mainly because of their dynamic range and the complexity, a reason why further subsampling of the tissue of interest might be helpful (Werner and Jahn, 2010). One study focussed on mitochondria in gray matter (GM), which suggested dysfunction in the mitochondrial respiratory chain in MS (Broadwater et al., 2011). In line with the findings of bulk transcriptomics data, proteins involved in inflammation and demyelination were upregulated in MS, but to find new disease mechanisms that can be targeted for therapies, more specific and sensitive techniques are needed. However, proteomics has been elaborately applied in mouse models of MS and the results from these studies may be worth trying to validate in humans.

Metabolomics is a relatively newly termed “omics” approach to systemically study metabolites in a sample, and the first metabolomics studies in MS were performed in the 1990s (Lynch et al., 1993). This is useful as metabolites are usually the end product of a biological process allowing us to conclude function. Despite the novelty of this approach, it has already found wide usage in MS and in its animal models, to try to identify biomarkers in body fluids like CSF, blood, and urine. With this, it might also be possible to observe metabolites in different patients and respond to their individual needs by different drugs, which would be the first step to personalized precision medicine (Bhargava and Calabresi, 2016; Del Boccio et al., 2016).

Techniques to study brain WM in bulk have greatly shaped understanding of WM diseases, but we now have the technology to examine pathological changes at a single cell level, gaining even deeper and new insights into these diseases.

### How Do Single-Cell Transcriptomic Techniques Work?

Single-cell RNA sequencing (scRNA-seq) is a novel technology using the same principle of capturing and sequencing mRNAs in bulk approaches within a tissue, however with the improvement that individual mRNAs can be associated with each cell of origin. This is particularly important for brain pathologies, where not all cell types are equally affected, for example in MS, where oligodendroglia are primarily lost. Although scRNA-seq is a relatively young technology—the first article was published in 2009 (Tang et al., 2009)—many different commercial techniques are already on the market and new ones are emerging at a rapid speed (Svensson et al., 2018; Chen et al., 2019), possibly faster than the publication of this review. Whilst all of them aim to give a snapshot of the transcriptome of individual cells, they are quite different in the technology achieving this. All of them have their advantages and disadvantages depending on the specific scientific question to be answered, so choosing the right technique is an important step in the experimental design. For this, we should consider the number of cells available, the capture efficiency, transcriptome coverage, and cost per cell. Several reviews have summarized and compared single-cell RNA-seq technologies (Haque et al., 2017; Picelli, 2017; Svensson et al., 2018; Chen et al., 2019). Despite the high number of technologies, from an experimental point of view, there are two approaches: studying a high number of cells at a lower resolution (up to tens of thousands of cells) or studying a low number of cells (generally <1,000 cells) at a higher resolution.

The first approach generally uses droplet-based technologies (Macosko et al., 2015; Zheng et al., 2017), whilst the second approach mainly uses well- or device-based technologies (Figures 2A,B) to capture single cells (Picelli et al., 2014; Hagemann-Jensen et al., 2020). Droplet-based methods use unique molecular identifiers (UMIs) and/or barcodes to label individual cells and mRNAs during the initial steps, so that the library preparation can be performed in bulk, rather than creating individual libraries in wells. As the droplet-based methods are aimed for high throughput, the costs per cell are much cheaper in comparison to well-based technologies. Also with this barcoding approach, copy numbers of mRNAs within a cell can at least in theory directly be measured, without the need of using additional standards such as External RNA Control Consortium (ERCC) spike-ins (Baker et al., 2005). To keep the sequencing costs at a realistic level, usually only the 3’ or the 5’ ends of the mRNA are amplified and sequenced, which only allows information of whether a gene is expressed or not, with a limited ability to examine splicing variants or SNPs. Conversely, with well- or device-based approaches, it allows the study of splice variants of genes, but the cost per cell is higher.

Sometimes, a combination of both techniques might be useful. Using unbiased droplet-based techniques to look at the entirety of cells in a tissue of interest helps to get an overview of all cells, including rare cell populations, and to find appropriate markers for these. This is especially useful in understudied tissues such as the human brain, as established markers for rodent cells are not always appropriate in humans. Cell populations of further interest, including rare populations or subpopulations, can then be studied at a deeper resolution using a full-length sequencing approach, after isolation or enrichment using the previously identified markers.

Currently, scRNA-seq experiments are cutting-edge and popular, generating much data and new hypotheses about the heterogeneity of cell function in all tissues with high impact publications. However, it is essential to validate these data and to keep the research question in mind, as sometimes a more classical approach will lead to an answer quicker, more easily, and cheaply. Although bulk approaches seem to be outdated at the moment, these technologies have also improved and are still important tools in studying human pathologies.

### What Are the Challenges and Drawbacks of Performing Human Single-Cell RNA-Seq?

All of these single-cell technologies were originally developed for cultured cells or rodent tissue, whilst their application to human tissue has only started to boom in recent years. Besides ethical constraints and the limited availability of human tissue (control equally as pathological tissue), there are more technical challenges that delayed the revolution in this field. Single-cell technologies were developed for viable single-cell suspensions, which is often not possible in WM pathologies, where most tissue is obtained post-mortem and not during biopsies. Hence, cell viability and tissue quality are often already below the accepted threshold to perform the experiments once the tissue arrives in the hands of the researcher. By this time, surviving cells may be highly selected and their transcriptome likely to have changed dramatically, with degraded RNA resulting in bad data quality and lower biological meaning. Moreover, to reduce confounding factors, all samples should be run ideally together or at least in as small a number of batches as possible (Hicks et al., 2017), which is impossible when using fresh biopsy or autopsy material, as individual tissue samples are sometimes only available months apart. For these reasons, most research so far in the human brain has used single-nuclei (sn) RNAseq using the same technologies from archived and frozen tissue samples instead of fresh viable cells. On comparing cells vs. nuclei, there is now the consensus that although nuclei generally yield a lower number of reads, they can add useful information on the biology. This tissue source may even protect from immediate changes in the transcriptome resulting from cell stress during cell isolation and the proportional representation of the *in vivo* situation may be more preserved, as, during live isolation, vulnerable cells are more likely to die resulting in their underrepresentation (Bakken et al., 2018; Wu et al., 2019). However, a new study suggested that using nuclei is not always a good alternative, as only highly abundant transcripts are detected and the more subtle changes related to the activation state of human microglia could not be distinguished (Thrupp et al., 2020). To get a clear picture of the advantages and disadvantages of using nuclei or cells, more comparative work using the same tissues, experimental setups, and sequencing depth will be helpful. However, given the high number of publications, it is already clear that snRNA-seq is an important tool to resolve biological questions, especially for human pathologies when no other tissue source is available.

More generally, once the decision has been made to use cells or nuclei, other challenges arise. Although at first sight, plentiful rich data is a clear advantage, it can also lead to data overload, that nobody knows what to do with. Especially with droplet-based technologies, individual sc/snRNA-seq experiments generate so many data points that research groups may only process a small part of it: for example, the experimental design might include all unselected nuclei, however, only a certain cell-type may be analyzed. Here is where open access sharing of these data is essential, allowing other groups to use these data to address their research questions and save a lot of time. This also allows for some mitigation of expense, as the rapid development of these technologies comes at a high cost. Although these techniques are becoming cheaper, the costs of commercial kits are still high and the cost per cell must be considered in the experimental plan. Homemade technologies are inevitably cheaper, but require knowledge and time to set up and may not be as robust and comparable between research labs as commercial ones. The costs for the sequencing should not be forgotten, which often equals the cost of the cell capture and cDNA library preparation. The depth of the experimental analysis obtained depends on the depth of sequencing.

A major limitation of sc/snRNA-seq is the number of transcripts that can be detected within a cell. Although the captured transcripts are often treated as representing the entirety of the transcriptome, in reality only about 5–20% of the transcripts are captured depending on the method, leaving about 80% of the biology undiscovered (Islam et al., 2014; Ding et al., 2020). These missing transcripts are usually ones with a low abundance that may represent more subtle changes between cell states. A recent advance is the development of a full-length sequencing method that reaches a significantly deeper transcriptome coverage per cell and thus results in a clearer separation of clusters (Hagemann-Jensen et al., 2020), but unfortunately, this method is not yet suitable for high throughput. Another limitation of these technologies comes from how RNA is captured. Most methods use oligos to capture polyadenylated mRNA only and especially droplet-based methods, additionally only use 3’ amplification. Few technologies have been developed to amplify the 5’ end of the RNA, however, the libraries are also prepared with the polyA-tail, still only accounting for the same type of RNA (Svensson et al., 2018; Chen et al., 2019). Other forms of biologically interesting RNAs, such as many microRNAs are not identifiable using this capture method and detection of splicing variants of genes is more limited (unless using full-length sequencing).

Once the sequencing data are generated, data storage is another often unconsidered problem. As datasets become bigger, in terms of sample number, cell number, and sequencing depth, the output data files become bigger and too large to be stored on a standard computer, instead of requiring big data servers or cloud storage, which come at a further cost. Data handling capacity challenges go hand in hand with increasing data size and depth, and analysis of these datasets generally requires a high-end workstation or, better, access to a computational cluster with the respective expertise. Associated with the fast development of scRNA-seq technologies, there is a large expansion in the available tools for data analysis, which are evolving all of the time and are generally open source and therefore free. This can give a bewildering variety of options on how to analyze the dataset. The challenge here is to find the right tool that is suitable for the data of interest, as not all available tools are. Helpful comparisons have emerged, for example in an overview of 45 current tools to calculate pseudotime trajectories (Saelens et al., 2019), as not all of them are equally suited for every dataset. Another example would be the availability of different clustering methods, with Seurat (Satija et al., 2015), Monocle (Trapnell et al., 2014), and Conos (Barkas et al., 2019) as highly used examples. Each of them uses a different algorithm and clustering approach, as outlined in Duò et al. (2018), and might thus result in different final clusters of which all may be valid. These are only examples, but every step in the experiment and the analysis part has many options. One study outlined this problem and showed that using only a minimum of options in different steps and combining it differently already results in ~3,000 different pipelines for analysis (Vieth et al., 2019). This field is as experimental as wet lab work, and it may be useful to use several analysis tools purporting to do the same thing on one dataset, to determine how robust the analysis is. However, ultimately, the only way to discover if the analysis is correct is to validate the results using other methods.

### What Have We Learned About WM Pathologies Using Single-Cell/Nuclei Transcriptomic Approaches?

Pioneering scRNA-seq analysis in rodent brain tissue clearly showed the detection of all brain cells that were distinguishable by specific markers (Zeisel et al., 2015). Of interest to MS, a key study in mouse oligodendrocytes (Marques et al., 2016) first used scRNA-seq to report their heterogeneity suggesting different inherent functions of oligodendrocytes not only between the brain and the spinal cord but even in the same region of the brain. The first studies using snRNA-seq on normal human brain tissue were proof of principle that this method was suitable in such tissue and that there is cellular and regional heterogeneity (Habib et al., 2017; Lake et al., 2018) and were the starting point of many following studies. Not surprisingly, it did not take long before this technology was used to study brain pathologies including MS (Figure 4).

With this hitherto unreached resolution, cellular heterogeneity in MS tissue was demonstrated in oligodendrocytes, neurons, microglia and astrocytes (Jäkel et al., 2019; Masuda et al., 2019; Schirmer et al., 2019; Wheeler et al., 2020) with disease-specific cell types or different heterogeneous states present in different proportions in MS compared to controls. In their study, Jäkel et al. (2019) found heterogeneous oligodendroglial states in non-pathological brain tissue and contrary to the current idea that all oligodendrocytes in MS lesions are equally vulnerable, reported that some of these states were over- and some underrepresented. Although the functional role of this cellular heterogeneity is not yet clear, this skew in the proportions of different oligodendrocyte states seen in MS was present in both NAWM and in MS lesions, again adding to the evidence that NAWM is indeed not normal, as previously shown for microglia (van der Poel et al., 2019). Furthermore, these data were able to identify a small population of previously unknown oligodendroglia with immunological functions (Falcão et al., 2018; Jäkel et al., 2019) which may influence disease pathogenesis as it suggests that oligodendrocytes may be an active player in the disease as well as a vulnerable target. This is of importance, as therapeutic approaches simply aiming to increase differentiation of oligodendrocytes to improve remyelination may need to be reconsidered, as replacing the “correct” type may be preferred. Another study using a similar approach to address the cellular composition of MS lesions found specific signatures for stressed oligodendrocytes, reactive astrocytes, and activated microglia, especially at the rim of demyelinated lesions. As this study also included cortical GM tissue, the authors reported a selective loss of CUX2-expressing upper layer excitatory projection neurons in the GM both in demyelinated and partially remyelinated lesions (Schirmer et al., 2019). In line with the previous study, they also found that some stressed oligodendrocytes seem to be capable of antigen presentation. This again confirms that damage does not affect all cells equally and that there is still a large gap of knowledge about disease mechanisms in MS. These studies are mostly descriptive, but a recent study in zebrafish has demonstrated that two distinct subgroups of oligodendrocyte precursor cells (OPCs) identified by scRNA-seq are confirmed to be functionally distinct with one primarily making networks and the other primarily differentiating into oligodendrocytes to make myelin (Marisca et al., 2020). Although this study was performed on normal zebrafish, this may also be important as if this is similar in humans, it may again force us to rethink our therapeutic remyelination strategies in MS, aiming to stimulate differentiating OPCs selectively. With a focus on microglia, Masuda et al. (2019) described microglial heterogeneity for the first time in the non-pathological human brain and additionally found clusters of disease-related microglia in MS patients that were similar to rodent animal models of MS, but with high inter-personal variability. A very recent study has directed its focus on astrocytes in MS showing that astrocytes in mice and humans are also heterogeneous. The authors found a clear MS-associated astrocyte cluster actively promoting CNS inflammation by the regulation of gene expression (Wheeler et al., 2020).

These technologies have also reached other human brain pathologies such as Alzheimer’s disease (AD; Grubman et al., 2019; Mathys et al., 2019; Zhou et al., 2020), Huntington’s disease (Al-Dalahmah et al., 2020) and other psychiatric disorders (Renthal et al., 2018; Velmeshev et al., 2019; Nagy et al., 2020). Although AD is usually considered a neuronal disease most of the GM, it has been surprisingly found that oligodendrocytes in the WM do show a significant transcriptional change in the disease adapting their metabolism to neuronal degeneration (Mathys et al., 2019; Zhou et al., 2020). OPCs also seem involved, as in AD, OPCs repress apolipoprotein E (APOE), which is a genetic risk factor for this disease, strengthening the hypothesis that oligodendroglia actively contribute to pathogenesis (Grubman et al., 2019). This study used the known AD risk genes to study how these contribute to disease in a cell-specific manner, as a relevant strategy to focus on the analysis of the wealth of data. Another recent study found that besides neurons, OPCs are majorly disturbed in major depressive disorder and this seemed to be coupled with their interaction with neurons rather than their ability to differentiate and myelinate (Nagy et al., 2020), which demonstrated that using this technology is important to disentangle the functions of subsets of cells. Most importantly, these new studies have started to shed new light on neurodegenerative and psychiatric diseases, moving away from a neurocentric view of these diseases with new recognition of the importance of glial cells in their pathogenesis—a shift in the research landscape.

### What Other Techniques Can We Use to Complement the sc/snRNAseq Approach?

RNA-seq at a single cell/nuclei level is only the start, with the fast development of other single-cell resolution technologies, including epigenetic methods. Assay for Transposase-Accessible Chromatin using sequencing (ATAC-seq) has already long been used to assess the bulk chromatin accessibility and the chromatin signature of cellular DNA (Buenrostro et al., 2013) including the human brain in health and disease (Corces et al., 2017; Bryois et al., 2018; Fullard et al., 2018), but can now also be done at the single-cell/nuclear level (Figure 2C). This adds information about the transcriptional regulation of different cell types and has already widely been used on rodents (Preissl et al., 2018; Sinnamon et al., 2019), but also human brain tissue (Zhong et al., 2020). A very recent study used this method to identify new single nucleotide polymorphisms (SNPs) with a more functional annotation than classically found with GWAS and found new risk-factors for Parkinson’s and AD (Corces et al., 2020). Bioinformatics tools are emerging to integrate scRNA-seq with scATAC-seq data to get a deeper understanding of the transcriptional and genomic landscape within one individual cell. Further explorations of the epigenomic landscape at a single cell level include DNA methylation profiling to detect methylation marks identifying regulatory programs in different cell populations (summarized in Fiers et al., 2018). It is already possible to detect epigenetic marks and gene expression in the same cell, not only bioinformatically but also experimentally, as shown with the sc-GEM (single-cell analysis of genotype, expression, and methylation) assay on cultured human fibroblasts (Cheow et al., 2016) and its use on human tissue would be another highly valuable method to understand its pathology.

Clearly, single-cell DNA/RNA changes can imply function but protein detection adds a further level of information to determine a cell’s behavior. Single-cell technologies have also entered the protein field with Cytometry by the time of flight (CyTOF), although this is not yet unbiased but requires a selection of markers of interest. This method uses metal-labeled antibodies to detect cellular antigens that are then analyzed by mass cytometry. This approach is similar to classical Fluorescence-activated cell sorting (FACS), but with a broader separation of metals which overcomes the limit of the overlap of fluorophores and allows the use of around 40 antibodies together (up to 100 when considering isotopes as well). With a bioinformatics analysis approach, high numbers of single cells can be thoroughly profiled. This approach has been used to characterize a change in the populations of peripheral immune cells of MS patients (Böttcher et al., 2019a) as well as to characterize multiple different region-dependent populations of microglia in the human brain that are distinguishable from peripheral cells (Böttcher et al., 2019b). CyTOF can also directly be applied to histological tissue sections—called imaging mass cytometry—and can be used to profile individual cells whilst maintaining the spatial information. Although still a fledgling technique at the spatial level, this has already successfully been applied in MS brain tissue to characterize astrocytes and peripheral cells in MS lesions (Park et al., 2019) and to characterize the immune cell landscape within different lesions from an individual MS patient (Ramaglia et al., 2019).

For a disease such as MS, spatial information is very important, due to the focal nature of demyelinated lesions, but there may also be pathological changes in more restricted areas in other neurodegenerative pathologies as well, e.g., in AD. Although not yet at a single-cell resolution, spatial transcriptomics technologies aim to capture the whole transcriptome of each of very small areas of a tissue section in an unbiased way in combination with histological analysis (Ståhl et al., 2016). Using the same principle of capturing and barcoding mRNA as droplet-based methods, this technology allows the location of the origin of an individual mRNA to a defined spot on a predefined grid on which the tissue has been placed, thus maintaining the spatial information. This technology is already being used on human tissue (Maynard et al., 2020), and with an earlier version in ALS (Gregory et al., 2020). With the clear advantage of capturing the transcriptome at a high resolution whilst maintaining spatial information, spatial transcriptomics technologies are clearly at the forefront of development and may in the future be more widely used than current sc/snRNA-seq technologies. They are either based on sequencing the transcripts *in situ* after having been barcoded (Ke et al., 2013; Lee et al., 2014; Wang et al., 2018; Gregory et al., 2020; Lundin et al., 2020; Maynard et al., 2020), or use highly multiplexed single molecular fluorescent *in situ* hybridization probes detectable using confocal microscopy (Lubeck et al., 2014; Shah et al., 2016). The current limitation of sequencing-based methods is the low detection of transcripts. Multiplexed *in situ* methods on the other hand are restricted by the number of probes (hundreds to thousands) due to the limited availability of fluorophores and optical resolution of individual molecules, making them less suitable for an unbiased discovery-driven research approach. However, recent developments have combined the methods, using sequential hybridization with *in situ* sequencing to theoretically cover the whole transcriptome with only a few fluorophores (Shah et al., 2016; Eng et al., 2019). As a result, a higher number of transcripts per cell can be detected. Although these methods have not yet been implemented on human brain tissue, which will be challenging due to its high autofluorescence, this high resolution of individual mRNAs will not only allow the localization of cells within tissue but also will allow the study of the subcellular localization of mRNAs, clear advantages in comparison to scRNA-seq methods. Unfortunately, these methods do only work well on thin tissue sections, limiting the information we gain from a three-dimensional point of view. Sequencing-driven spatial methods in particular are still expensive and are thus tend to be performed on small tissue pieces with few sections from an individual, which may introduce some bias to the biological findings.

Validation of results, preferably on a separate cohort of tissue, is essential by classical immunohistochemistry/fluorescence and *in situ* hybridization, and/or these burgeoning multiplexing technologies, mentioned above. These have allowed spatially detection of 100 different transcripts by *in situ* sequencing (Lundin et al., 2020) or around 100 proteins and 1,000 genes using oligonucleotide labeling in a tissue section (Geiss et al., 2008; Kulkarni, 2011). Although imaging mass cytometry is usually used to characterize novel cell populations, it can clearly serve as a validation method for transcriptomic data as well. These require analysis tools to distinguish signals in different cells, but appear very useful and are likely to become standard to address human pathologies in the future.

### What Does the Future Hold?

The outputs of all of these technologies applied to human WM pathologies are still no more than descriptive pathology—although on a much deeper level than was ever possible before and at least implying function. This work, however, is just the start of a new era of single-cell resolution techniques that will revolutionize human pathology and will most likely become a standard technology for pathological assessment. The richness of these data will allow us to take the next step, which is to address more functional changes to gain a deeper understanding of the diseases. For example, snRNA-seq will allow us to study transcriptomic changes in a high number of cells in many different types of MS lesions which then may allow us to reclassify them on a functional level, namely their regenerative potential rather than using the classical degenerative description. Moreover, these data may allow us to determine lesion markers that can be used for PET-imaging and will thus be an invaluable tool for disease diagnosis, prognosis, and response to therapies. Furthermore, as most of the data are gained similarly and deposited with its metadata on open-source databases, it is then easier to compare many brain regions (such as WM and GM, or brain and spinal cord) or diseases with each other, to gain a much clearer picture of the cellular architecture of our brain. The Human Cell Atlas is a collaborative effort to exactly achieve this aim^1^ not only for the brain but for the entire human body.

So far, most of these technologies are used individually by different groups but in the future, complementary but different technologies will be used in the same experimental setup, and their outputs integrated, as recently shown from the Allen Brain Institute (Bakken et al., 2020). Maybe in 10 years from now, it will be possible to look at the transcriptome, the epigenome, the proteome and the metabolome on a single cell level from the same tissue source at once, as suggested in Figure 3. Attempts to achieve this have already been made in recent preprint manuscripts where the authors were able to simultaneously study either proteins and mRNA (Vistain et al., 2020) or chromatin accessibility and gene expression (Ma et al., 2020) in single cells. This will allow us to look at the same data from many different perspectives to gain a deeper understanding of individual cells in health and disease and further explore the pathological mechanism. The options here seem endless with money as the only limit!

## Conclusion

Analysis and understanding of human WM pathologies have come a long way from being purely descriptive histological analysis to gaining cell type-specific information at a single-cell resolution (Figure 4). This development goes hand in hand with the development of novel technologies, although their use in human tissue is inevitably more challenging than in animal models. We can now sequence the RNA of a cell, examine the state of the chromatin and current proteomic tools will likely become more sensitive as well, which will allow us to explore the proteome of individual cells. Although all of these technologies have developed separately, we will need to link them together to see the full picture in the future. This will allow us to gain a deeper insight into human pathologies and will become important tools not only for basic science, but will also revolutionize diagnostics and may pave the way for developing new therapies. However, even though these technologies are developing at an exciting and rapid speed, it is important to retain a clear focus on the research question to be answered, to avoid distraction by an ocean of data and high costs, and to ensure that we advance biology.

## Author Contributions

SJ and AW wrote and edited the manuscript. SJ also designed and prepared the figures.

## Conflict of Interest

The authors declare that the research was conducted in the absence of any commercial or financial relationships that could be construed as a potential conflict of interest.

## Footnotes

^1^https://www.humancellatlas.org/
